# Peripheral Groups of Dicationic Pyrazinoporphyrins Regulate Lipid Membrane Binding

**DOI:** 10.3390/membranes12090846

**Published:** 2022-08-30

**Authors:** Daria A. Polivanovskaia, Anna N. Konstantinova, Kirill P. Birin, Valerij S. Sokolov, Oleg V. Batishchev, Yulia G. Gorbunova

**Affiliations:** 1 Frumkin Institute of Physical Chemistry and Electrochemistry, Russian Academy of Sciences, 31/4 Leninskiy pr., 119071 Moscow, Russia; 2 Kurnakov Institute of General and Inorganic Chemistry, Russian Academy of Sciences, 31 Leninskiy pr., 119991 Moscow, Russia

**Keywords:** photosensitizers, porphyrins, lipid membrane, adsorption, singlet oxygen, styryl dyes

## Abstract

Photodynamic therapy (PDT) is a widely used technique for skin cancer treatment and antimicrobial therapy. An improvement in PDT efficiency requires not only an increase in quantum yield of photosensitizer (PS) molecules but also their applicability for biological systems. Recently, we demonstrated that the activity of porphyrin-based PSs in the lipid membrane environment depends on the nature of the cation in the macrocycle due to its interactions with the lipid phosphate moiety, as well as the orientation of the PS molecules inside the membrane. Here, we report the synthesis, membrane binding properties and photodynamic efficiency of novel dicationic free-base, Ni(II) and Zn(II) pyrazinoporphyrins with terminal tetraalkylammonium units (**2H-1**, **Ni-1** and **Zn-1**), to show the possibility to enhance the membrane binding of PS molecules, regardless of the central cation. All of these substances adsorb at the lipid membrane, while free-base and Zn(II) porphyrins actively generate singlet oxygen (SO) in the membranes. Thus, this study reveals a new way to tune the PDT activity of PSs in biological membranes through designing the structure of the peripheral groups in the macrocyclic photosensitizer.

## 1. Introduction

Photosensitizers (PSs) represent a class of molecules capable of generating reactive oxygen species (ROSs), including singlet oxygen (SO), upon irradiation. This ability of photosensitizers allows them to be used in photodynamic therapy (PDT)—a method of killing pathogenic cells and microorganisms, widely used in the treatment of skin cancer [1,2,3,4] and antimicrobial therapy, including fighting against multidrug-resistant bacteria [5,6,7]. It is noteworthy that PSs for antimicrobial therapy cover a wider range of suitable molecules since they do not demand excitation in red and infrared regions, such as for cancer treatment. Applicability of PSs for PDT, as well as the enhancement of their photodynamic efficiency, require their activity in biological systems and physiological conditions. Recently, we developed a new approach for the investigation of the efficiency of PSs at the bilayer lipid membrane (BLM) based on measurements of boundary potentials at the membrane/water interface [8,9,10]. Utilizing this approach, we demonstrated that for monocationic β-imidazolium-substituted porphyrins, the nature of the central cation in the macrocyclic ligand has a significant influence on both the efficiency of the lipid membrane binding of the photosensitizer and its photodynamic output, as revealed through the degradation of target molecules (TMs) by SO [8]. The highest photodynamic efficiency is detected for the In(III) complex, while Zn(II) and free-base PS molecules demonstrate both a lower rate of oxidation for TMs and a weaker membrane binding. 

In the present study, we raised the question of whether it would be possible to increase the photodynamic efficiency of the heterocycle-substituted porphyrin and its Zn(II) complex through changing the nature of peripheral groups. The development of approaches for the introduction of *N*-heterocyclic fragments to the periphery of the porphyrin macrocycle attracted synthetic efforts by our research group [11,12,13]. Such five- and six-membered fragments may serve as convenient spacers, gathering a porphyrin macrocycle with one or two functional moieties. At the same time, we succeeded to develop new approaches for the formation of functionalized porphyrins containing imidazole and pyrazine units [14,15,16,17,18,19]. As a result, we designed a representative of a new type of hydrophilic dicationic photoactive porphyrin derivatives, namely, pyrazinoporphyrins, bearing terminal alkylammonium units.

Thus, in the present work, we report further developments in a synthesis of cationic porphyrin derivatives and an analysis of their lipid membrane binding and a photodynamic efficiency in a lipid environment. We demonstrate that not only interactions of the central metal atom with a lipid phosphate moiety, but also the structure of the peripheral groups of the PS molecule may regulate its activity in biological membranes.

## 2. Materials and Methods

All the used reagent-grade chemicals were purchased from commercial suppliers, unless otherwise stated. The solvents were purified according to conventional methods [20]. The starting 2,3-diamino-tetrakis(4-butoxyphenyl)porphyrinato nickel(II) was prepared following the published procedure [15]. Chromatographic purification was performed using Silica 60 0.063–0.2 mm (Macherey-Nagel, Duren, Germany) and neutral alumina (Macherey-Nagel, Duren, Germany). Merck aluminum plates (TLC Silica 60 F254) were used for TLC analyses with hexane/dichloromethane mixtures as eluents. Size-exclusion chromatography was performed using Bio-Beads SX-1 (Bio-Rad Laboratories, Hercules, USA) sorbent with CHCl_3_:MeOH eluent (98:2).

MALDI-TOF mass spectra were recorded using a Ultraflex (Bruker Daltonics, Bremen, Germany) spectrometer in the positive ion mode without a matrix. UV–Vis spectra were recorded using a Evolution 201 spectrophotometer (Thermo Scientific, Waltham, MA, USA) in rectangular quartz cells with 0.1–10 mm optical path in 250–900 nm range. ^1^H NMR spectra were recorded using a Avance III spectrometer (Bruker, Felanden, Swizerland) with 600 MHz proton frequency in CDCl_3_, or the mixture CDCl_3_/MeOD, at an ambient temperature with the use of the residual solvent resonance as an internal reference.

4-(3-bromopropyloxy)-benzaldehyde was prepared following the published procedure [21] except for the application of 1,3-dibromopropane instead of 1-bromo-3-chloropropane. The product was obtained as yellow oil with 93% purity containing 4-allyloxybenzaldehyde as a contaminant, with a yield of 60%.

^1^H-NMR (CDCl_3_; δ, ppm; ^n^*J*, Hz): 9.89 (s, 1H, CHO), 7.84 (d, 2H, ^3^*J* = 8.3, CH_Ar_), 7.01 (d, 2H, ^3^*J* = 8.4, CH_Ar_), 4.20 (t, 2H, ^3^*J* = 5.8, CH_2_O), 3.61 (t, 2H, ^3^*J* = 6.4, CH_2_Br), 2.36 (quint, 2H, ^3^*J* = 6.1, CH_2_). Distinct signals of the minor allyl derivative were observed at 6.05 (ddt, 1H, ^3^*J* = 16.2, 10.5, 5.3), 5.43 (d, 1H, ^3^*J* = 17.3), 5.33 (d, 1H, ^3^*J* = 10.5), 4.63 (d, 2H, ^3^*J* = 5.3, CH_2_O).

**Ni-3:** The starting Ni(II) 2-nitro-3-aminoporphyrinate (153 mg, 0.15 mmol) was dissolved in a mixture of DCM (45 ml) and MeOH (1.65 ml) under argon. 10% Pd/C (75 mg) and NaBH_4_ (86 mg, 2.25 mmol) were subsequently added, and the resulting mixture was vigorously stirred at ambient temperature for ca. 15 min until the complete consumption of the starting material was detected via TLC (DCM/hexane = 2:1). Afterwards, the mixture was filtered through Celite-545 under argon then immediately evaporated, and the residue was dissolved under argon in o-dichlorobenzene (20 ml). 4-(3-bromopropyloxy)-benzaldehyde (366 mg, 1.5 mmol) was added to the above solution in o-dichlorobenzene (4 ml) and TsOH (5.2 mg, 20 mol%) was added to the reaction mixture upon stirring. The mixture was heated at 100 °C overnight and evaporated to dryness. The residue in DCM was applied to the column packed with silica in hexane and eluted with hexane/DCM mixtures (0 → 50% of DCM). The fractions containing the target pyrazinoporphyrin were repurified with size-exclusion chromatography. The evaporation of these fractions yielded 177 mg of a 68:32 mixture of the target **Ni-3** and a side product according to NMR containing 3-bromopropyl and allyl moieties. The corresponding yields were 123 mg (57%) and 54 mg (27%).

Repeated chromatographic purification of a sample of Ni(II) bis-bromopropyl-pyrazinoporphyrinate using silica gel and Bio-Beads SX-1 allowed the obtainment of the target compound with a maximal purity of 80%. The spectral characteristics represented below correspond to this sample.

^1^H NMR (CDCl_3_; δ, ppm; ^n^*J*, Hz): 8.83 (d, 2H, ^3^*J* = 4.9, H_β_), 8.75 (d, 2H ^3^*J* = 4.9, H_β_), 8.71 (s, 2H, H_β_), 7.89 (d, 4H, ^3^*J* = 8.2, *o*-H_Ar_), 7.82 (d, 4H, ^3^*J* = 8.1, *o*-H_Ar_), 7.42 (d, 4H, ^3^*J* = 8.6, *o*-H_Pz-Ar_), 7.21 (t, 8H, ^3^*J* = 8.9, *m*-H_Ar_), 6.80 (d, 4H, ^3^*J* = 8.6, *m*-H_Pz-Ar_), 4.24 (t, 4H, ^3^*J* = 6.5, ^Bu^CH_2_O), 4.20 (t, 4H, ^3^*J* = 6.5, ^Bu^CH_2_O), 4.15 (t, 4H, ^3^*J* = 5.8, ^Pr^CH_2_O), 3.65 (t, ^3^*J* = 6.4, 3H, ^Pr^CH_2_Br), 2.36 (quint, 4H, ^3^*J* = 6.1, ^Pr^CH_2_), 2.02 (quint, 4H, ^3^*J* = 6.4, ^Bu^CH_2_), 1.93 (quint, 4H, ^3^*J* = 6.3, ^Bu^CH_2_), 1.71 (sext, 4H, ^3^*J* = 7.5, ^Bu^CH_2_), 1.64 (sext, 4H, ^3^*J* = 7.5, ^Bu^CH_2_), 1.13 (t, 6H, ^3^*J* = 7.4, CH_3_), 1.08 (t, 6H, ^3^*J* = 7.4, CH_3_).

Contaminating allyl substituent: 6.11 (ddt, 1H, ^3^*J* = 16.2, 10.6, 5.3), 5.46 (d, 1H, ^3^*J* = 17.2), 5.33 (d, 1H, ^3^*J* = 10.5 Hz,), 4.58 (d, 2H, ^3^*J* = 5.2 Hz, CH_2_).

UV–Vis (CHCl_3_; λ, nm (log ε)): 261 (4.46), 294 (4.43), 349 (4.39), 390 (4.53), 442 (5.32), 546 (4.29), 579 (3.88).

MALDI-TOF MS: calcd. for C_80_H_78_Br_2_N_6_NiO_6_ [M]^+^ 1434.37, found 1434.26.

**Ni-1: Ni-3** (19 mg, 13 μmol) was dissolved in CHCl_3_ (6 ml) and Et_3_N (2ml). The mixture was refluxed overnight, cooled to an ambient temperature and evaporated. The residue was dissolved in CHCl_3_, applied to the column packed with neutral alumina in CHCl_3_ and eluted with CHCl_3_/MeOH mixtures (0 → 4% of MeOH). The fractions containing the target dicationic material were evaporated and repurified with size-exclusion chromatography which provided 9 mg (42%) of the pure **Ni-1** compound.

^1^H NMR (CDCl_3_; δ, ppm; ^n^*J*, Hz): 8.81 (d, 2H, ^3^*J* = 4.9, H_β_), 8.75 (d, 2H, ^3^*J* = 4.9, H_β_), 8.72 (s, 2H, H_β_), 7.88 (d, 4H, ^3^*J* = 8.1, *o*-H_Ar_), 7.80 (d, 4H, ^3^*J* = 8.1, *o*-H_Ar_), 7.34 (d, 4H, ^3^*J* = 8.3, *o*-H_Pz-Ar_), 7.20 (d, 4H, ^3^*J* = 8.3, *m*-H_Ar_), 7.18 (d, 4H, ^3^*J* = 8.2, *m*-H_Ar_), 6.75 (d, 4H, ^3^*J* = 8.3, *m*-H_Pz-Ar_), 4.20 (m, 12H, ^Bu^CH_2_O + ^Pr^CH_2_O), 3.63 (br.t, 4H, ^3^*J* = 8.4, PrCH_2_N), 3.53 (br.q, 12H, ^3^*J* = 7.3, ^Et^CH_2_), 2.28 (br.s, 4H, ^Pr^CH_2_), 1.95 (m, 12H, ^Bu^CH_2_), 1.66 (m, 4H, ^Bu^CH_2_), 1.42 (t, 18H, ^3^*J* = 7.2, ^Et^CH_2_), 1.09 (t, 12H, ^3^*J* = 7.4, ^Bu^CH_3_).

^13^C NMR (CDCl_3_; δ, ppm): 159.23, 159.21, 158.46, 148.42, 147.27, 144.23, 142.60, 141.76, 134.71, 134.33, 133.30, 132.91, 132.87, 132.75, 132.64, 132.16, 131.92, 131.57, 120.37, 116.29, 113.91, 113.21, 113.14, 68.11, 67.98, 64.18, 54.96, 53.63, 31.84, 31.63, 22.52, 19.66, 19.52, 14.25, 14.07, 8.02.

UV–Vis (CHCl_3_; λ, nm (log ε)): 289 (4.30), 347 (4.28), 391 (4.39), 442 (5.21), 546 (4.15), 580 (3.75).

**2H-3:** The mixture of **Ni-3** and the corresponding allyl relative (68:32, 177 mg) were dissolved in TFA (3.45 ml), and H_2_SO_4_ (0.86 ml) was added subsequently upon stirring at ambient temperature. The mixture was vigorously stirred for 3 min, diluted with CHCl_3_ (10 ml) and quenched with a solution NaOAc × 3H_2_O (13.2 g) in water (35 ml). The mixture was stirred for 15 min, transferred to a separating funnel and the organic layer was separated. The organic phase was further washed with water (50 ml), saturated with aqueous NaHCO_3_ (50 ml) and evaporated. The residue was applied to the column packed with silica in hexane and eluted with hexane/DCM mixtures (0→100% of DCM). The evaporation of the fractions provided 95 mg of the target **2H-3** (94% purity), which corresponded to a 76% yield.

^1^H NMR (CDCl_3_; δ, ppm; ^n^*J*, Hz): 8.99 (d, 2H, ^3^*J* = 4.9, H_β_), 8.96 (d, 2H, ^3^*J* = 4.9, H_β_), 8.77 (s, 2H, H_β_), 8.12 (d, 4H, ^3^*J* = 8.1, *o*-H_Ar_), 8.06 (d, 4H, ^3^*J* = 8.2, *o*-H_Ar_), 7.43 (d, 4H, ^3^*J* = 8.4, *o*-H_Pz-Ar_), 7.28 (d, 4H, ^3^*J* = 8.2, *m*-H_Ar_), 7.26 (d, 4H, ^3^*J* = 8.4, *m*-H_Ar_), 6.79 (d, 4H, ^3^*J* = 8.4, *m*-H_Pz-Ar_), 4.28–4.23 (m, 8H, ^Bu^CH_2_O), 4.14 (t, 4H, ^3^*J* = 5.7, ^Pr^CH_2_O), 3.66 (t, 4H, ^3^*J* = 6.4, CH_2_Br), 2.37 (quint, 4H, ^3^*J* = 6.1, ^Pr^CH_2_), 2.03 (quint, 4H, ^3^*J* = 6.4, ^Bu^CH_2_), 1.98 (quint, 4H, ^3^*J* = 6.4, ^Bu^CH_2_), 1.71 (m, 8H, ^Bu^CH_2_), 1.16 (t, 6H, ^3^*J* = 7.4, CH_3_), 1.12 (t, 6H, ^3^*J* = 7.4, CH_3_), −2.60 (s, 2H, NH).

^13^C NMR (CDCl_3_; δ, ppm): 159.23, 159.12, 159.00, 155.29, 149.49, 147.73, 145.59, 139.86, 138.50, 135.60, 135.12, 134.35, 134.28, 134.05, 132.75, 131.90, 128.04, 127.89, 121.01, 117.62, 113.70, 112.84, 112.77, 68.06, 67.95, 65.25, 32.54, 31.90, 31.60, 29.97, 19.69, 19.46, 14.16, 14.01.

UV–Vis (CHCl_3_; λ, nm (log ε)): 271 (4.51), 292 (4.48), 381 (4.59), 436 (5.59), 526 (4.35), 567 (3.88), 598 (3.97), 657 (3.34).

MALDI-TOF MS: calcd. for C_80_H_80_Br_2_N_6_O_6_ [M^+^] 1378.45, found 1278.16.

**2H-1: 2H-3** (94% purity, 46 mg, 33μmol) was dissolved in dry CHCl_3_ (10 ml) and Et_3_N (2 ml). The mixture was refluxed under argon overnight, cooled to an ambient temperature and evaporated. The residue was dissolved in CHCl_3_, applied to the column packed with neutral alumna in CHCl_3_ and eluted with CHCl_3_/MeOH mixtures (0 → 6% of MeOH). The obtained fraction of the target dicationic material was subsequently purified with size-exclusion chromatography which afforded 19 mg (37%) of the pure **2H-1**.

^1^H NMR (CDCl_3_; δ, ppm; ^n^*J*, Hz): 8.95 (d, 2H, ^3^*J* = 5.0, H_β_), 8.94 (d, 2H, ^3^*J* = 4.9, H_β_), 8.73 (s, 2H, H_β_), 8.06 (d, 4H, ^3^*J* = 7.9, *o*-H_Ar_), 7.97 (d, 4H, ^3^*J* = 7.9, *o*-H_Ar_), 7.31 (d, 4H, ^3^*J* = 8.2, *o*-H_Pz-Ar_), 7.23 (d, 4H, ^3^*J* = 8.0, *m*-H_Ar_), 7.17 (d, 4H, ^3^*J* = 7.9, *m*-H_Ar_), 6.69 (d, 4H, ^3^*J* = 8.2, *m*-H_Pz-Ar_), 4.20 (t, 4H, ^3^*J* = 6.5, ^Bu^CH_2_O), 4.16 (t, 4H, ^3^*J* = 6.4, ^Bu^CH_2_O), 4.07 (t, 4H, ^3^*J* = 5.4, ^Pr^CH_2_O), 3.59–3.48 (m, 4H, ^Pr^CH_2_N), 3.38 (q, 12H, ^3^*J* = 7.2, ^Et^CH_2_), 2.13 (br.s, 4H, ^Pr^CH_2_), 1.97–1.89 (m, 8H, ^Bu^CH_2_), 1.71–1.58 (m, 8H, ^Bu^CH_2_), 1.31 (t, 18H, ^3^*J* = 7.2, ^Et^CH_3_), 1.07 (m, 12H, ^Bu^CH_3_), −2.65 (s, 2H, NH).

^13^C NMR (CDCl_3_; δ, ppm): 159.28, 159.23, 158.33, 155.44, 149.52, 147.96, 145.50, 139.86, 138.58, 135.68, 135.17, 134.28, 134.22, 134.17, 133.02, 132.05, 128.18, 128.05, 121.20, 117.70, 113.80, 112.96, 112.88, 68.12, 67.94, 64.06, 54.77, 53.49, 31.85, 31.64, 22.42, 19.67, 19.51, 14.26, 14.06, 7.89.

UV–Vis (CHCl_3_; λ, nm (log ε)): 272 (4.43), 379sh (4.54), 435 (5.57), 526 (4.27), 566 (3.81), 598 (3.91), 657 (3.33).

MALDI-TOF MS: calcd. for C_86_H_94_N_7_O_6_ [M-Et_3_N-H]^+^ 1320.73, found 1320.09.

**Zn-1:** The porphyrin **2H-3** (94% purity, 27 mg, 20 μmol) was dissolved in a mixture of CHCl_3_ (20 ml) and MeOH (5 ml), and Zn(OAc)_2_ (30 mg, 0.16 mmol) was added. The mixture was stirred at ambient temperature for 3 h and evaporated to dryness. The residue was redissolved in CHCl_3_ (10 ml) and Et_3_N (1 ml) was added. The obtained solution was refluxed under argon overnight, cooled to ambient temperature and evaporated. The residue was dissolved in CHCl_3_, applied to the column packed with neutral alumna in CHCl_3_ and eluted with CHCl_3_/MeOH mixtures (0 → 6% of MeOH). The obtained fraction of the target dicationic material was subsequently purified with size-exclusion chromatography which afforded 6 mg (28%) of the pure **Zn-1**.

^1^H NMR (20% MeOD in CDCl_3_; δ, ppm; ^n^*J*, Hz): 8.90 (d, 2H, ^3^*J* = 4.6, H_β_), 8.88 (d, 2H, ^3^*J* = 4.6, H_β_), 8.84 (s, 2H, H_β_), 8.08 (d, 4H, ^3^*J* = 8.2, *o*-H_Ar_), 8.02 (d, 4H, ^3^*J* = 8.1, *o*-H_Ar_), 7.44 (d, 4H, ^3^*J* = 8.4, *o*-H_Pz-Ar_), 7.26 (d, 4H, ^3^*J* = 8.2, *m*-H_Ar_), 7.25 (d, 4H, ^3^*J* = 8.1, *m*-H_Ar_), 6.76 (d, 4H, ^3^*J* = 8.4, *m*-H_Pz-Ar_), 4.30 (t, 4H, ^3^*J* = 6.4, ^Bu^CH_2_O), 4.26 (t, 4H, ^3^*J* = 6.4, ^Bu^CH_2_O), 4.08 (t, 4H, ^3^*J* = 5.4, ^Pr^CH_2_O), 3.43–3.36 (m, 4H, ^Pr^CH_2_N), 3.26 (q, 12H, ^3^*J* = 7.3, ^Et^CH_2_), 2.13–2.06 (m, 4H, ^Pr^CH_2_), 2.04–1.98 (m, 4H, ^Bu^CH_2_), 1.98–1.92 (m, 4H, ^Bu^CH_2_), 1.72 (sext, 4H, ^3^*J* = 7.4, ^Bu^CH_2_), 1.65 (sext, 4H, ^3^*J* = 7.5, ^Bu^CH_2_), 1.32 (t, 18H, ^3^*J* = 7.4, ^Et^CH_3_), 1.12 (t, 6H, ^3^*J* = 7.4, ^Bu^CH_3_), 1.09 (t, 7H, ^3^*J* = 7.4, ^Bu^CH_3_). 

UV–Vis (CHCl_3_; λ, nm (log ε)): 391sh (4.56), 444 (5.56), 563 (4.42), 601 (3.84).

**Experiments with bilayer lipid membranes (BLMs):** Bilayer lipid membranes (BLMs) were formed with the Mueller–Rudin technique [22] from the solution of 1,2-diphytanoyl-*sn*-glycero-3-phosphatidylcholine (Avanti Polar Lipids, Alabaska, AL, USA) in n-decane (Sigma-Aldrich, Saint Louis, MO, USA) in concentration of 15 mg/ml. The Teflon cell contained a septum with a round aperture (diameter of approximately 0.8 mm) separating two compartments of equal volume (2 ml each) which were continuously stirred using magnetic stirrers. Compartments were filled with buffer solutions of KCl (Sigma-Aldrich, Saint Louis, MO, USA), and *N*-(2-Hydroxyethyl)piperazine-*N*′-(2-ethanesulfonic acid), HEPES (Sigma-Aldrich, Saint Louis, MO, USA), in double-distilled water. The formation of the lipid bilayer was controlled through measuring its capacitance, which increased during the thinning of the membrane and approached a steady-state value after the membrane formation. Electrical measurements were performed with the aid of a pair of Ag/AgCl electrodes with agar bridges. The bridges were composed of standard plastic pipette tips, the bottom part of which was filled with agarose gel prepared in 0.1 M KCl solution, and the remaining volume was filled with the same working buffer solution as in the cell. The change in the boundary potential difference across the membrane was measured with the inner field compensation (IFC) method based on the detection of the second harmonics of the capacitance current [23]. This method allowed the measuring of the difference in boundary potentials (Δϕ_b_) between the two sides of the BLM after the addition of the porphyrins into one compartment of the cell and their adsorption at one side of the membrane. The equipment used for these measurements was similar to that described in [24]. 

The measuring cell was equipped with two windows: one for the optical monitoring of the formation of the BLM, and the other one for the illumination of the membrane with a semiconductor laser (wavelength 405 nm, optic power 1 mW). The porphyrins and target molecule 6-[2-(*N*,*N*-Dibutylamino)naphthyl]ethenyl-4′-pyridinium propanesulfonate, di-4-ANEPPS (Sigma-Aldrich, Saint-Louis, MO, USA), were added into the opposing compartments of the cell from the stock solutions in ethanol. The total concentration of ethanol in the water never exceeded 3%. The porphyrins were added into the compartment far from the light source to prevent the attenuation of the light beam due to its absorption when passing through the water solution.

The absorption spectra of porphyrins in the ethanol and water solutions were measured using a Panorama Fluorat 02 (Lumex, Saint Petersburg, Russia) fluorescence spectrophotometer. 

## 3. Results and Discussion

### 3.1. Synthesis

**Scheme 1:**
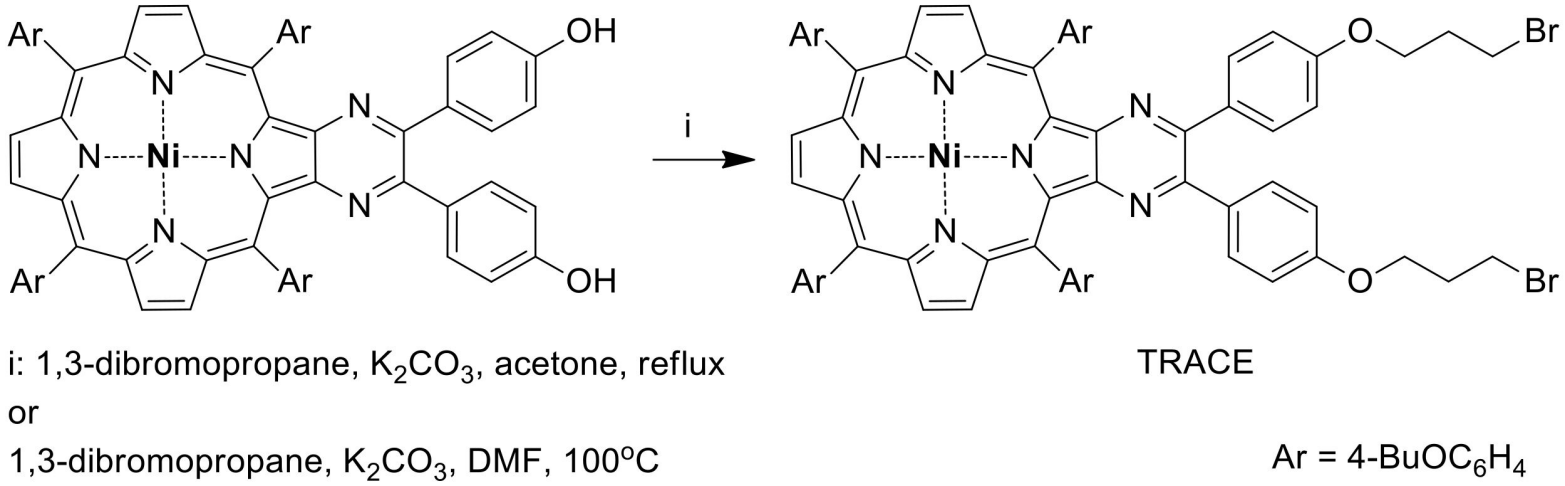
Alkylation of bis(4-hydroxyphenyl)pyrazinoporphyrin.

Recently, we reported the comprehensive investigation of the synthetic strategy for the preparation of porphyrins fused with five- [11,12,17] and six-membered heterocycles [14,15,18]. The developed methodology consisted of the condensation of labile and reactive 2,3-diaminoporphyrins with aromatic aldehydes under varied conditions. We revealed that the presence of the electron-donor substituents in the aromatic aldehyde, which are used for the condensation, possess drastic influence upon its reactivity and, thus, onto the yield of the prepared derivatives. In particular, the presence of the dimethylamino group virtually prevents condensation, while 4-hydroxybenzaldehyde still possesses sufficient reactivity to produce imidazo- and pyrazinoporphyrins [15]. Such precursors were expected to be convenient substrates for the alkylation with aliphatic dihalogenides for the introduction of peripheral reactive sites with further quaternization (Scheme 1). Unexpectedly, the treatment of bis(4-hydroxyphenyl)-substituted pyrazinoporphyrin with 1,3-dibromopropane under typical alkylation conditions provided the desired product only in trace amounts. 

**Scheme 2:**
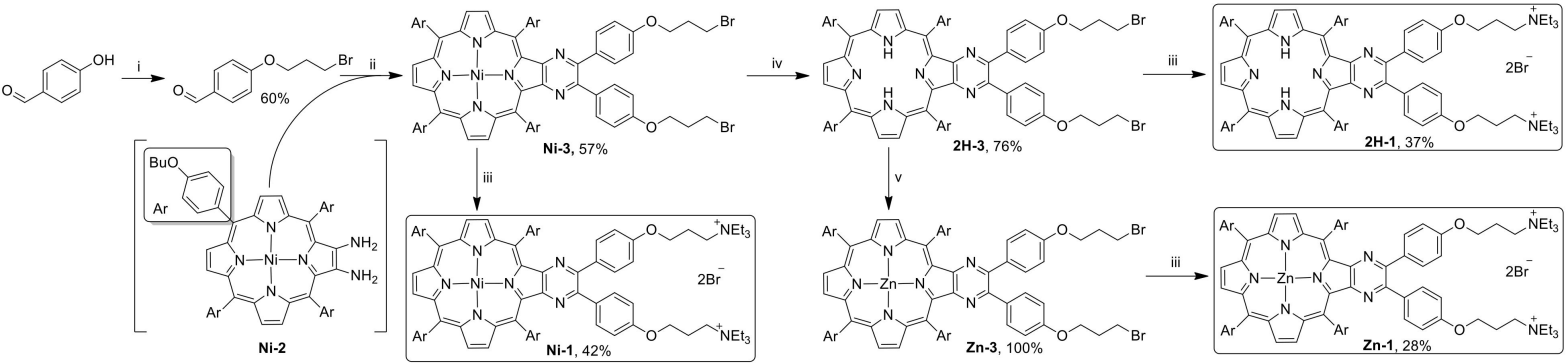
Synthesis of dicationic derivatives **Ni-1**, **2H-1** and **Zn-1**. *i*: 1,3-dibromopropane, K_2_CO_3_, MeCN, reflux; *ii*: 10 equiv. of aldehyde, 20 mol% TsOH, DCB, 100 °C; *iii*: Et_3_N, CHCl_3_, reflux; *iv*: H_2_SO_4_, TFA; *v*: Zn(OAc)_2_, CHCl_3_, MeOH.

In this respect, a different strategy for the synthesis of the required dicationic pyrazinoporphyrin was proposed (Scheme 2). Porphyrin **2H-1** was selected for the investigation along with the corresponding zinc(II) **Zn-1** and nickel(II) **Ni-1** complexes. Ni(II) and Cu(II) ions are known to quench the photophysical properties of porphyrins completely through the fast irradiative relaxation of the excited states [25]. However, in the present study, we decided to use Ni(II) as a model for the evaluation of the membrane binding efficiency of the peripheral groups. First, 4-(3-bromopropyloxy)-benzaldehyde was prepared through selective alkylation of 4-hydroxybenzaldehyde. The obtained product contained ca. 7% of inseparable allyloxybenzaldehyde formed under basic alkylation conditions via the elimination of the terminal bromine atom. The condensation of the functional aldehyde with nickel(II) 2,3-diaminoporphyrin **Ni-2** provided the corresponding disubstituted pyrazinoporphyrin **Ni-3**, which in turn was successfully demetalated to give **2H-3** and further converted into the zinc(II) complex **Zn-3**. Interestingly, the ratio of the allyloxy to the γ-bromopropyloxy substituent increased upon condensation, and the contaminating allyl-substituted porphyrin was also inseparable from the target one. In this respect, the series of bis(bromopropyl) porphyrin derivatives was further subjected to quaternization through treatment using trimethylamine as a mixture. It is worth mentioning that the prolonged reflux in CHCl_3_ was required for the complete conversion of the starting bromoalkyl derivatives, and the yield of the target dicationic porphyrins remained low. It was found, for **Ni-1** as an example, that a decrease in the reaction temperature through changing CHCl_3_ to CH_2_Cl_2_ prevented the substitution, while an increase in the temperature through performing the reaction in 1,2-dichloroethane resulted in a further decrease in the yield. Nevertheless, the target compounds **2H-1**, **Ni-1** and **Zn-1** were successfully prepared with 37%, 42% and 28% yields of the quaternization stages. The final dicationic derivatives were successfully separated chromatographically from the monocationic by-products and, thus, obtained in a pure form.

**Figure 1:**
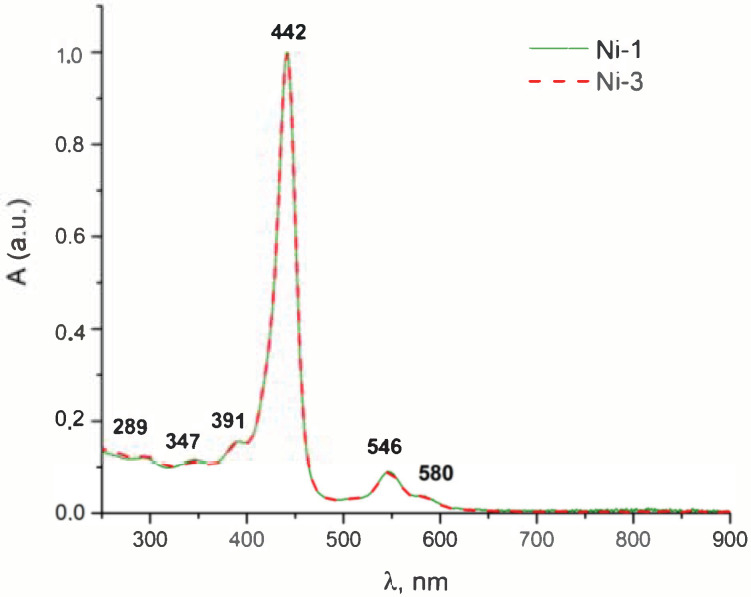
UV–Vis spectra of solution of **Ni-1** and **Ni-3** in CHCl_3_.

The structure and purity of compounds were proved by means of NMR, UV–Vis spectroscopy and mass spectrometry. The complete NMR and UV–Vis data for the target dicationic compounds **2H-1**, **Ni-1** and **Zn-1** are collectively represented in Supporting materials (Figures S1–S6 and S7–S9, respectively). It should be noted that the positions and shapes of the bands in the UV–Vis spectra are not sensitive to the presence of cationic fragments in the obtained molecules. Thus, the comparison of the spectra of **2H-1**, **Ni-1** and **Zn-1** with the spectra of the corresponding bromo precursors did not reveal considerable shifts of the typical bands (Figure 1). It allowed the conclusion of the vanishing influence of the ionic unit on the porphyrin electronic structure. It is also worth mentioning that butoxy groups provide sufficient solubility of **2H-1** and **Ni-1** in common organic solvents, e.g., CHCl_3_ and CH_2_Cl_2_. In contrast, **Zn-1** is virtually insoluble in such solvents, but its solutions in organic media can be obtained in the presence of MeOH. The hydrophilicity of obtained cationic compounds is discussed below.

**Figure 2:**
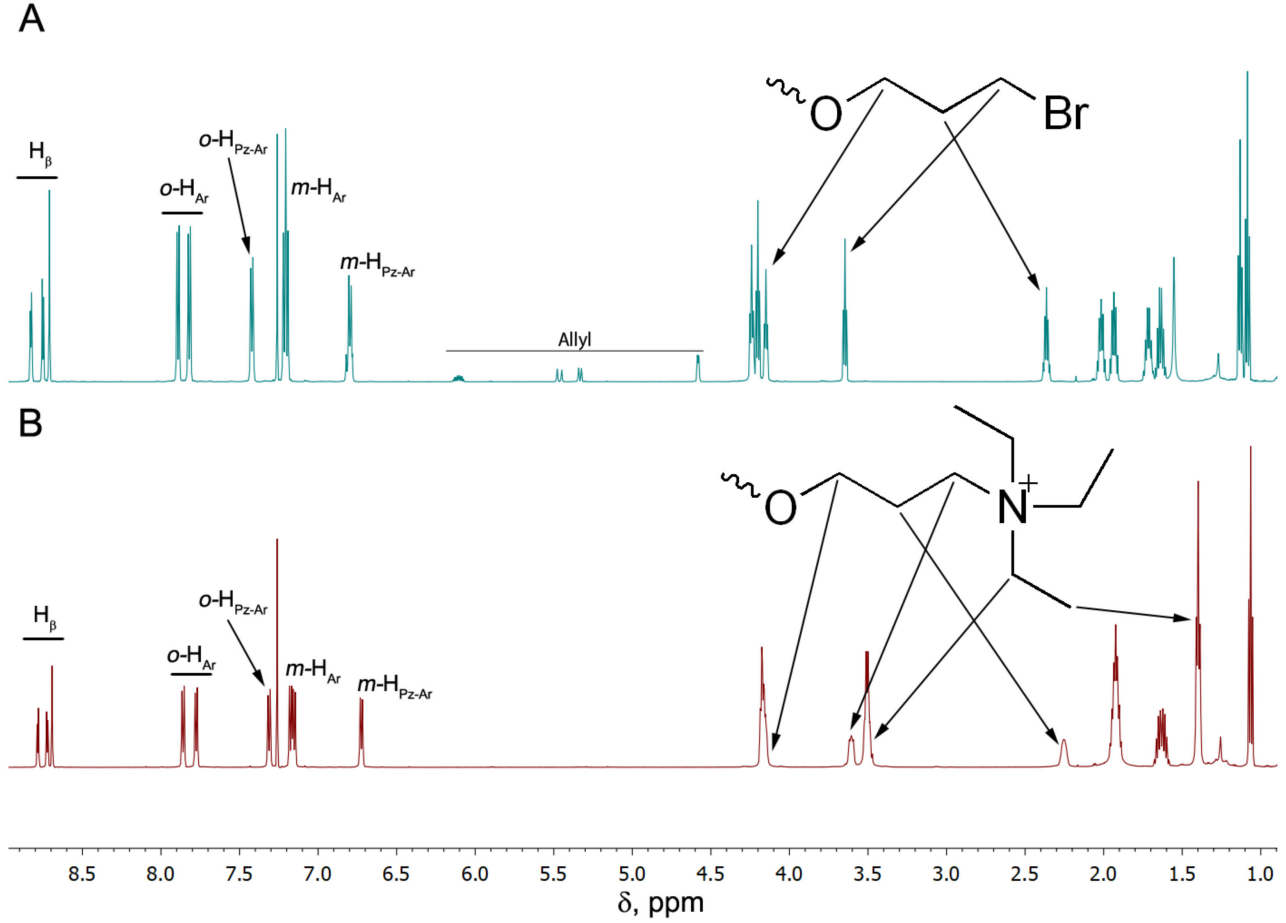
NMR spectra of **Ni-3** (**A**) and **Ni-1** (**B**) in CDCl_3_.

Surprisingly, despite the presence of a variety of alkyl chains in the molecule, their environment turned out to be sufficiently different for the interpretation of the NMR spectra of compounds (Figure 2). The introduction of the triethylammonium fragment instead of the bromine atom was clearly observed via the downfield shift of the signal associated with the corresponding CH_2_ group. It is noteworthy that the application of MALDI-TOF mass spectrometry was not informative for the dicationic derivatives, except **2H-1**. In the mentioned case, the fragmentation of the molecule was observed, and the peak in the spectrum corresponded to [M-Et_3_NH]^+^ which could be explained by the elimination from the Mannich-type fragment.

The aggregation of porphyrins is one of the major factors possessing significant influence upon their photophysical output. The aggregation of cationic porphyrins in water was reported by us previously [8]. This specific behavior of porphyrins depends on various structural and environmental factors. The ionic strength of the aqueous solutions is also a tool which allows the tuning of the properties of the ionic porphyrins. Thus, the variation in the ionic strength allowed the managing of the intercalation of the tetra(methylpyridinium)porphyrin with DNA, the process suppressed upon the increase in the NaCl concentration [26]. The suppression of the binding could be reasonably attributed to the aggregation of the porphyrin. Furthermore, the presence of the doubly charged cations in the medium resulted in a considerably larger decrease in the sensitizer efficiency compared to the mono-charged metal ions [27]. The length of the alkyl chain on the positively charged nitrogen atom also influenced the aggregation, which was enhanced with the extension of the chain [28]. Finally, the direct evidence of propensity of the tetraalkylammonium porphyrins for the dimerization was reported upon the increase in the NaCl concentration in the medium [29]. With these considerations, the influence of the ionic strength of the solutions on the aggregation of the porphyrins under discussion was estimated.

**Figure 3:**
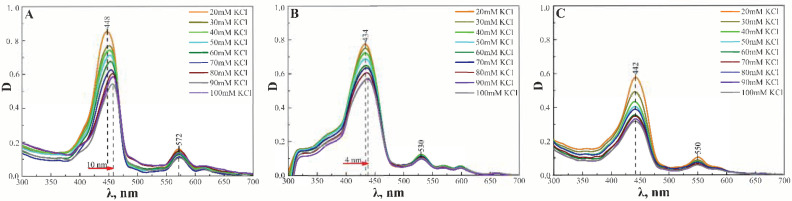
UV–Vis spectra of porphyrins in water solution (2mM HEPES, pH 7) upon increase in the concentrations of KCl for: (**A**) 4 μM of **Zn-1**; (**B**) 4 μM of **2H-1**; (**C**) 6 μM of **Ni-1**.

We studied the influence of the ionic strength of the applied aqueous solutions on the aggregation of pyrazinoporphyrins with the analysis of changes in the UV–Vis spectra of porphyrins upon increase in the concentration of KCl in the 20–100 mM range (Figure 3). In all cases, the decrease in the intensity of absorption bands was observed along with the increase in the KCl concentration. Moreover, the considerable shift in the Soret band was revealed in these series of spectra for **Zn-1** and **2H-1**. Such behavior revealed the notable aggregation of the prepared dicationic porphyrins with the increase in the ionic strength of the used buffer solutions that prevented their further application in the physiological concentration of salts. Therefore, the minimal possible concentration of KCl (20 mM) was used in further experiments with the BLM.

### 3.2. Membrane Binding and Photodynamic Efficiency

**Figure 4:**
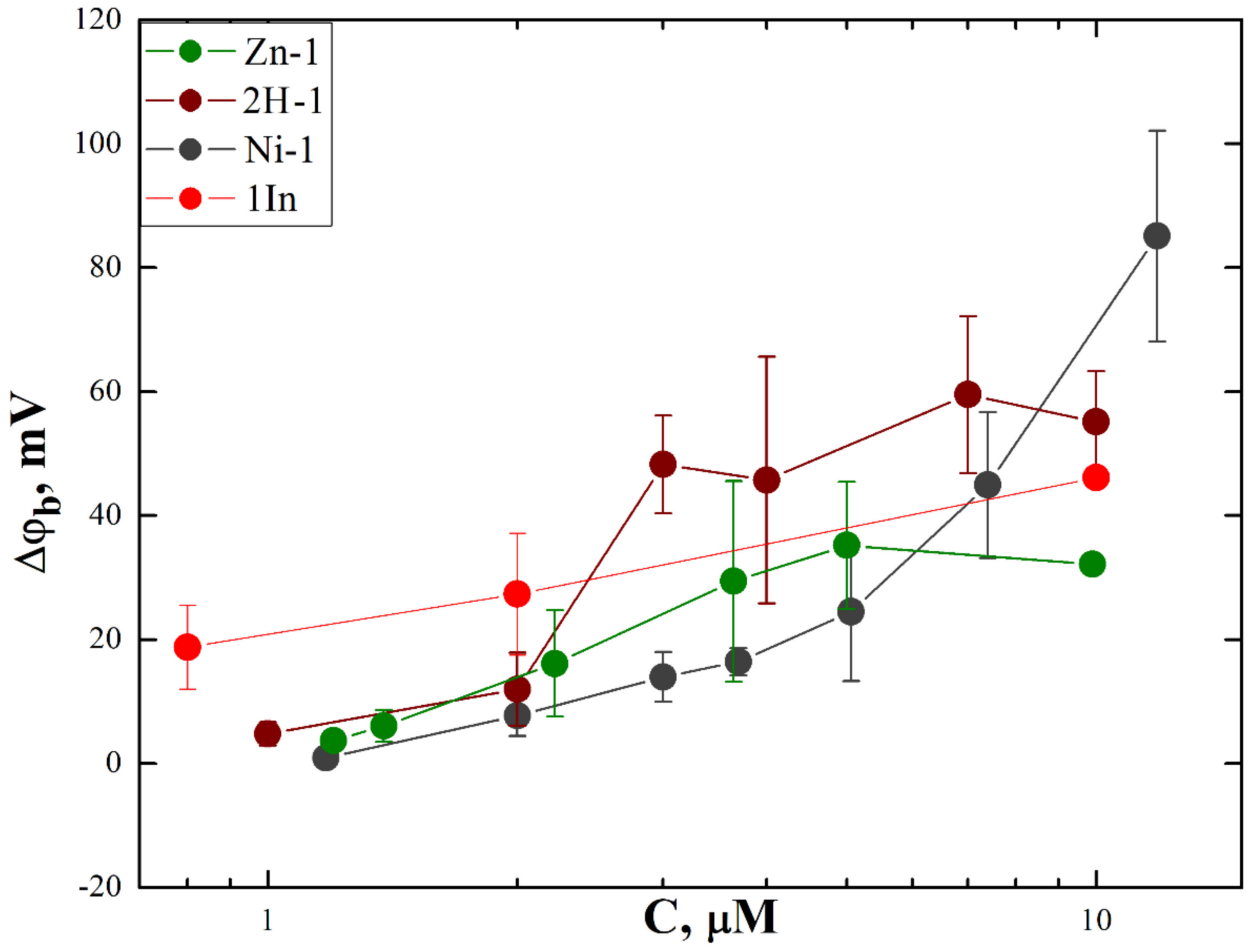
The change in the boundary potential difference across the BLM due to adsorption of porphyrins as a function of their concentration in the solution. Measurements were performed in buffer solutions containing 20 mM KCl, 2 mM HEPES, pH 7.0. Data for In(III) complex of β-imidazolium-substituted porphyrin (1In, red) were taken from [8] for the comparison.

The addition of ethanol solutions of porphyrins into the cell compartment at one side of the membrane led to a change in the boundary potential difference Δϕ_b_ measured with the IFC method. This indicated the adsorption of porphyrins at the BLM. The sign of the Δϕ_b_ corresponded to the adsorption of positively charged molecules that agreed with the structures of obtained porphyrins. The dependence of the Δϕ_b_ on the concentration of porphyrins added into the water solution is presented in Figure 4. To avoid the influence of the possible aggregation of porphyrins with time [8], in all experiments, we measured the change in Δϕ_b_ just after the single addition of the porphyrin solution with the given concentration. The comparison of three porphyrins showed maximal Δϕ_b_ values close to the In(III) complex of β-imidazolium-substituted porphyrin, which demonstrated the best membrane binding in our previous study [8]. This result suggested that the membrane binding of PS molecules was possibly regulated by not only the type of the central cation, but also the structure and nature of substituents in the porphyrin ring. The plateau of the dependencies for free-base porphyrin and its Zn(II) complex pointed to the possible aggregation of these PSs at high concentrations.

**Figure 5:**
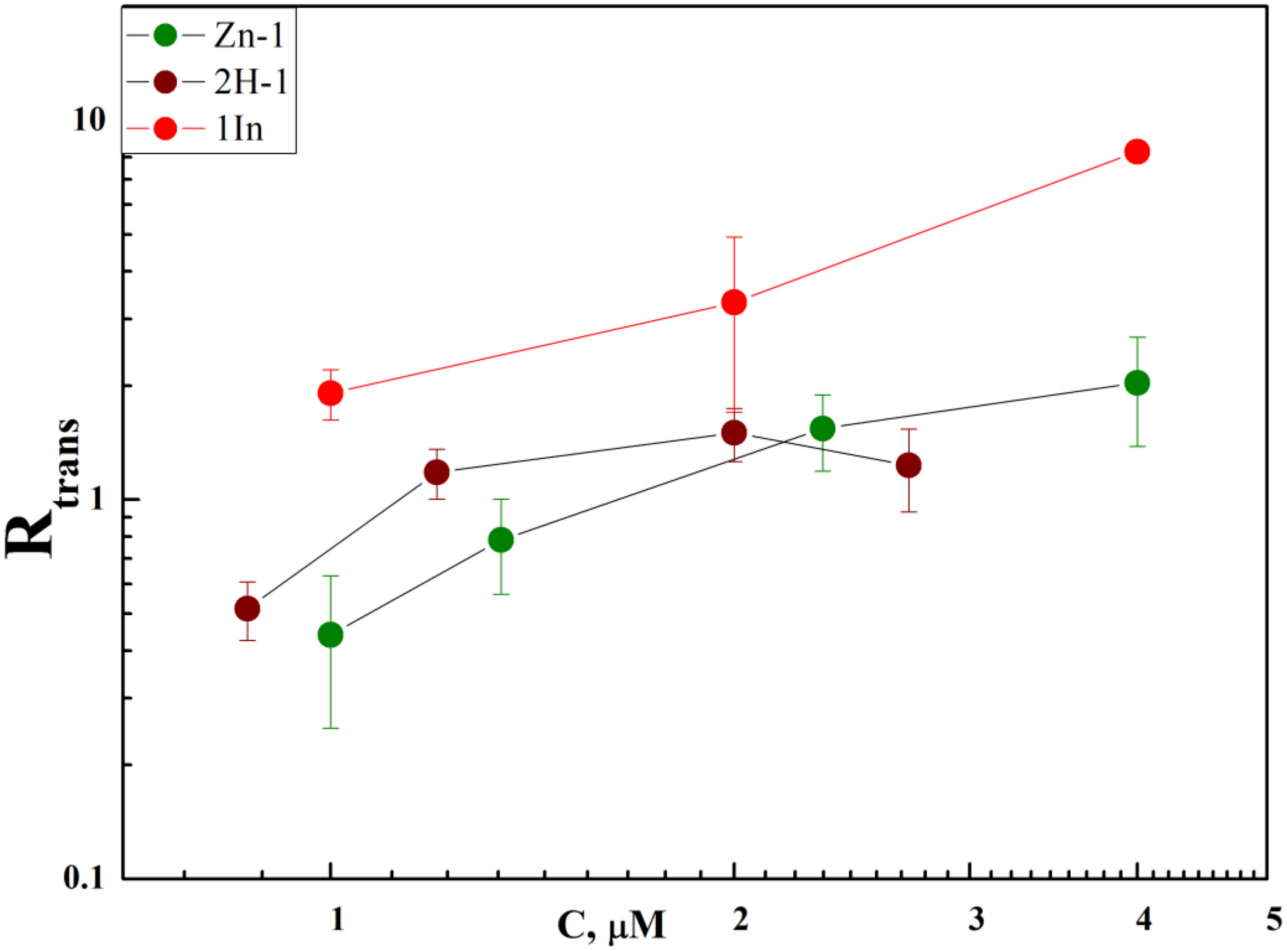
The dependence of the rate of oxidation of di-4-ANEPPS on the concentration of porphyrins in the water solution containing 20 mM KCl, 2 mM HEPES, pH 7.0. Data for In(III) complex of β-imidazolium-substituted porphyrin (1In, red) were taken from [8] for the comparison.

The photodynamic efficiency of the porphyrins was studied similarly to the early developed approach [24], through measuring the rate of oxidation of target molecules styryl dye di-4-ANEPPS (trap). This trap was added into the cell compartment at the opposite side to the one where the porphyrins were present to prevent the interaction between porphyrin and di-4-ANEPPS molecules in the water solution. The adsorption of di-4-ANEPPS molecules at the BLM resulted in a rise in the Δϕ_b_. The illumination of the BLM with both adsorbed di-4-ANEPPS and porphyrin molecules led to a decrease in the Δϕ_b_ value due to the oxidation of di-4-ANEPPS molecules. This photooxidation was observed for the free-base porphyrin and its Zn(II) complex. As mentioned above, the Ni(II) complex was not capable of generating SO. The oxidation rate, *R*, was calculated from the kinetics of the decrease in the Δϕ_b_ during the illumination and its restoration after switching the light off [24,27,28,29,30]:

(1) $$R=\frac{1}{\tau_{L}}-\frac{1}{\tau_{D}},$$

where *τ_L_* and *τ_D_* are the time constants of exponents approximating the kinetics of ϕ(t) during illumination and the following dark phase, respectively. The dependence of *R* on the concentration of porphyrins in the water solution is presented in Figure 5.

As follows from Figure 5, the rate *R* increased with the concentration of porphyrins. The comparison of the values of *R* for Zn(II) and the free-base pyrazinoporphyrins demonstrated that the photodynamic efficiency of these two compounds were close. However, the oxidation rate did not reach the values of the In(III) complex of β-imidazolium-substituted porphyrin, which demonstrated the best membrane binding in our previous study [8], and remained at the same level as the Zn(II) complex and free-base β-imidazolium-substituted porphyrins. Hence, peripheral groups did not change the photodynamic efficiency of the PS molecules. 

## 4. Conclusions

In the present work, we synthesized representatives of a new type of hydrophilic cationic porphyrins suitable for the PDT application in biological systems. Comparing pyrazinoporphyrins bearing terminal tetraalkylammonium units with β-imidazolium-substituted porphyrins, we demonstrated that cell membrane binding of PSs could be attenuated by their peripheral groups, in contrast to the previous findings, that it resulted only from the interactions of the central cation with lipid phosphate groups [8]. However, this type of binding did not change the rate of oxidation of the target molecules by generated SO. Nevertheless, increased membrane binding resulted in the higher surface concentration of PSs with the same level of these molecules in solution, thus, possibly providing higher efficiency of these molecules for PDT for the given bulk concentration.

## Data Availability

All the data is available on demand.

## Supplementary Materials

The following supporting information can be downloaded at: https://www.mdpi.com/article/10.3390/membranes12090846/s1, Figure S1: ^1^H NMR spectrum of **2H-1** (CDCl_3_); Figure S2: ^1^H-^1^H COSY spectrum of **2H-1** (CDCl_3_); Figure S3: ^1^H NMR spectrum of **Ni-1** (CDCl_3_); Figure S4: ^1^H-^1^H COSY spectrum of **Ni-1** (CDCl_3_); Figure S5: ^1^H NMR spectrum of **Zn-1** (20% MeOD in CDCl_3_); Figure S6: ^1^H-^1^H COSY spectrum of **Zn-1** (20% MeOD in CDCl_3_); Figure S7: a series of UV–Vis spectra of **2H-1** in CHCl_3_ (C = 2.6×10^−5^–2.4×10^−7^ M); Figure S8: a series of UV–Vis spectra of **Ni-1** in CHCl_3_ (C = 2.1×10^−5^–1.4×10^−6^ M); Figure S9: a series of UV–Vis spectra of **Zn-1** in CHCl_3_ (C = 1.9×10^−5^–1.8×10^−7^ M).
