# Supplementary material for: Peripheral Groups of Dicationic Pyrazinoporphyrins Regulate Lipid Membrane Binding
Source: Membranes (Basel). 2022 Aug 30;12(9):846. doi: 10.3390/membranes12090846 (PMC9505865; doi:10.3390/membranes12090846)
Supplement: Supplementary file 1 [file membranes-12-00846-s001.zip › membranes-1897943-supplementary.pdf]

Supplementary information

Peripheral Groups of Dicationic Pyrazinoporphyrins Regulate Lipid Membrane Binding

Daria A. Polivanovskaia [1], Anna N. Konstantinova [1], Kirill P. Birin [1], Valerij S. Sokolov [1], Oleg V. Batishchev [1] and Yulia G. Gorbunova [1,2*]

1 Frumkin Institute of Physical Chemistry and Electrochemistry, Russian Academy of Sciences, 31/4 Leninskiy pr., 119071 Moscow, Russia

2 Kurnakov Institute of General and Inorganic Chemistry, Russian Academy of Sciences, 31 Leninskiy pr., 119991 Moscow, Russia

* Correspondence: yulia@igic.ras.ru

NMR spectra of the dicationic compounds

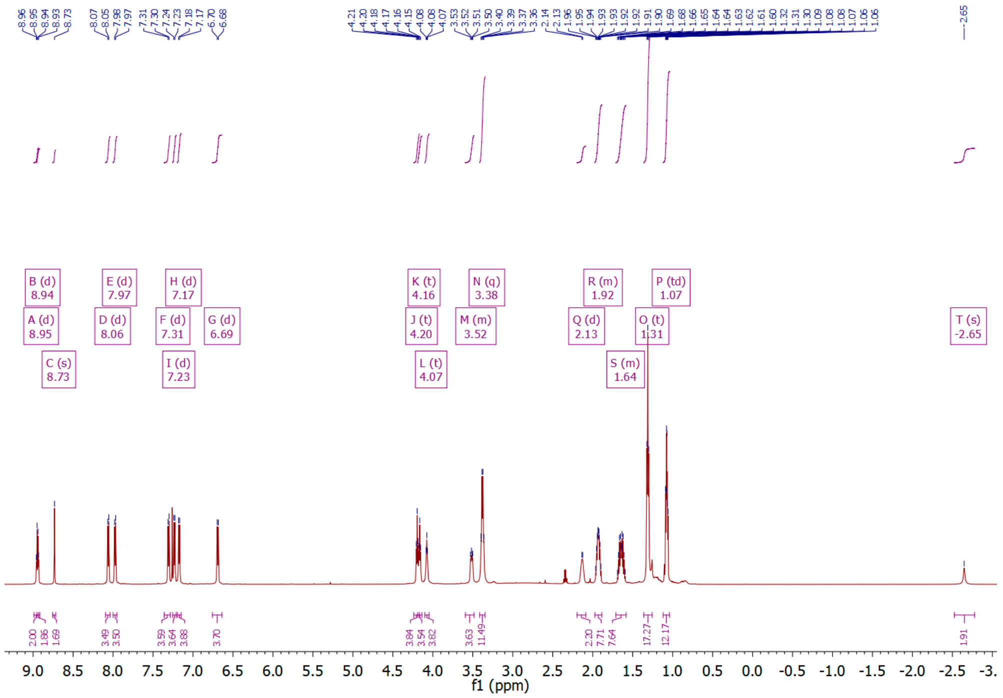

Figure S1. ^{1}H NMR spectrum of **2H-1** ($CDCl_3$).

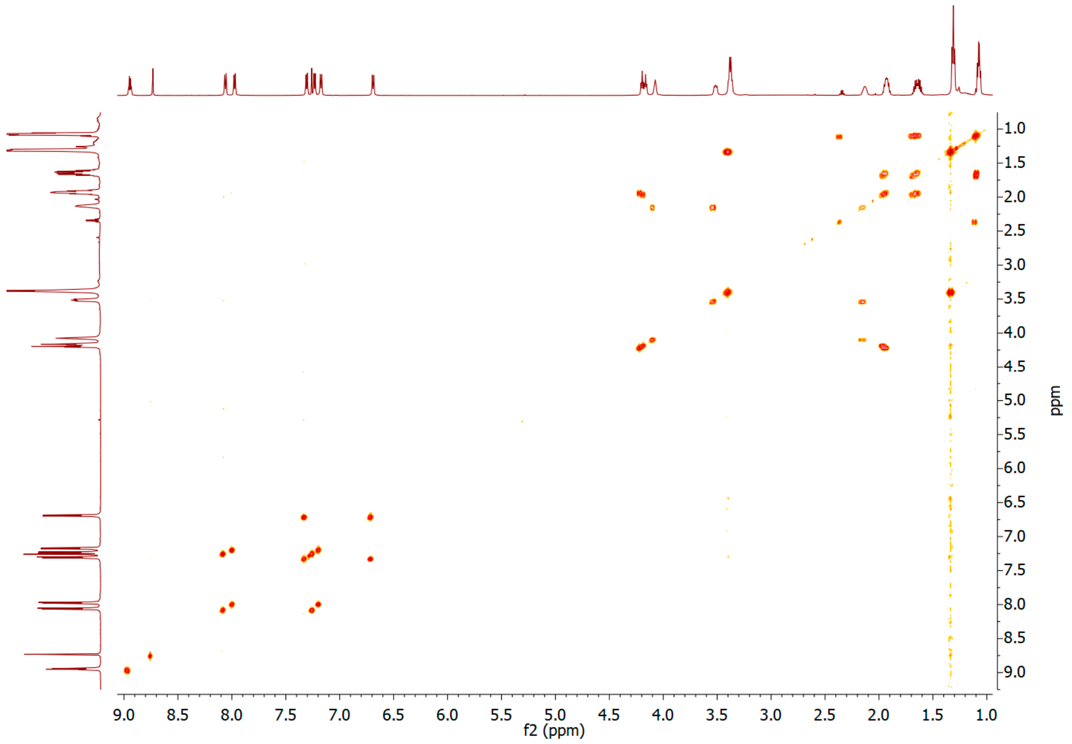

Figure S2. ^{1}H-^{1}H COSY spectrum of **2H-1** ($CDCl_3$).

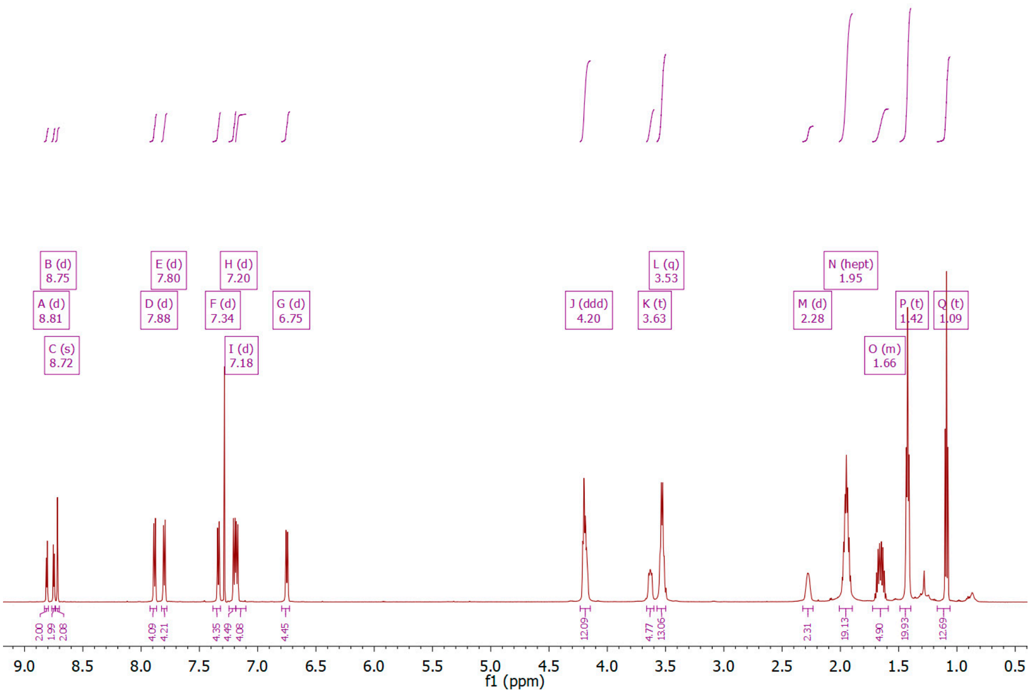

Figure S3. ^{1}H NMR spectrum of **Ni-1** ($CDCl_3$).

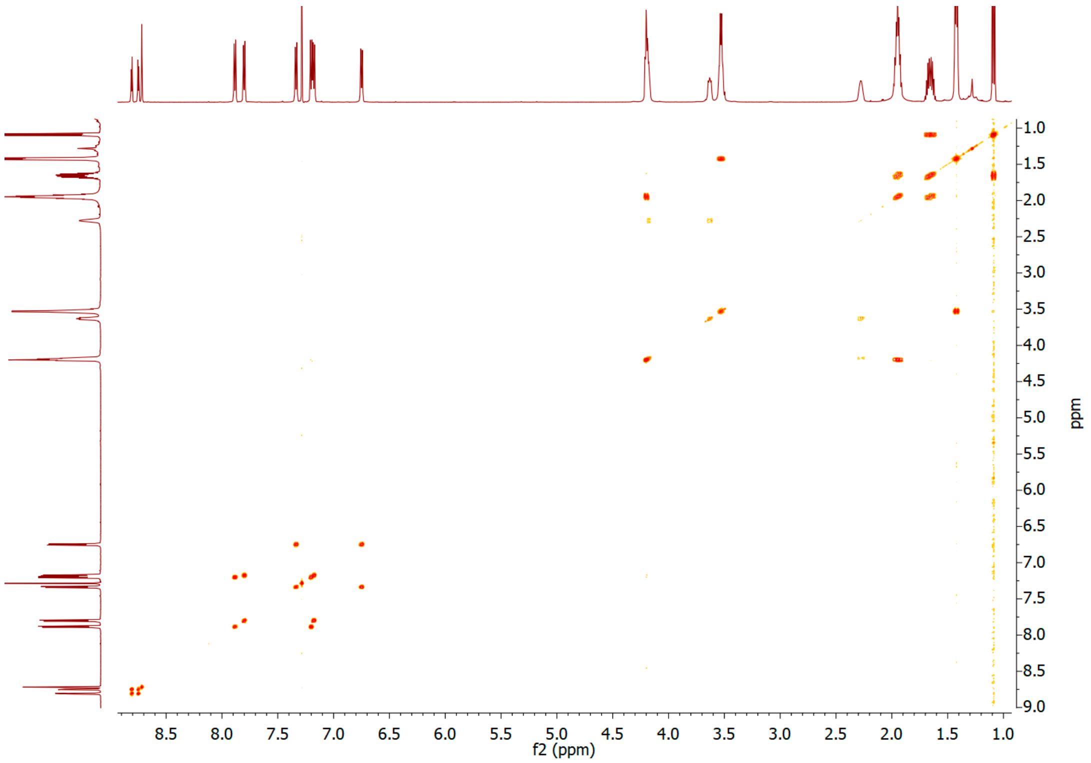

Figure S4. ^{1}H-^{1}H COSY spectrum of **Ni-1** ($CDCl_3$).

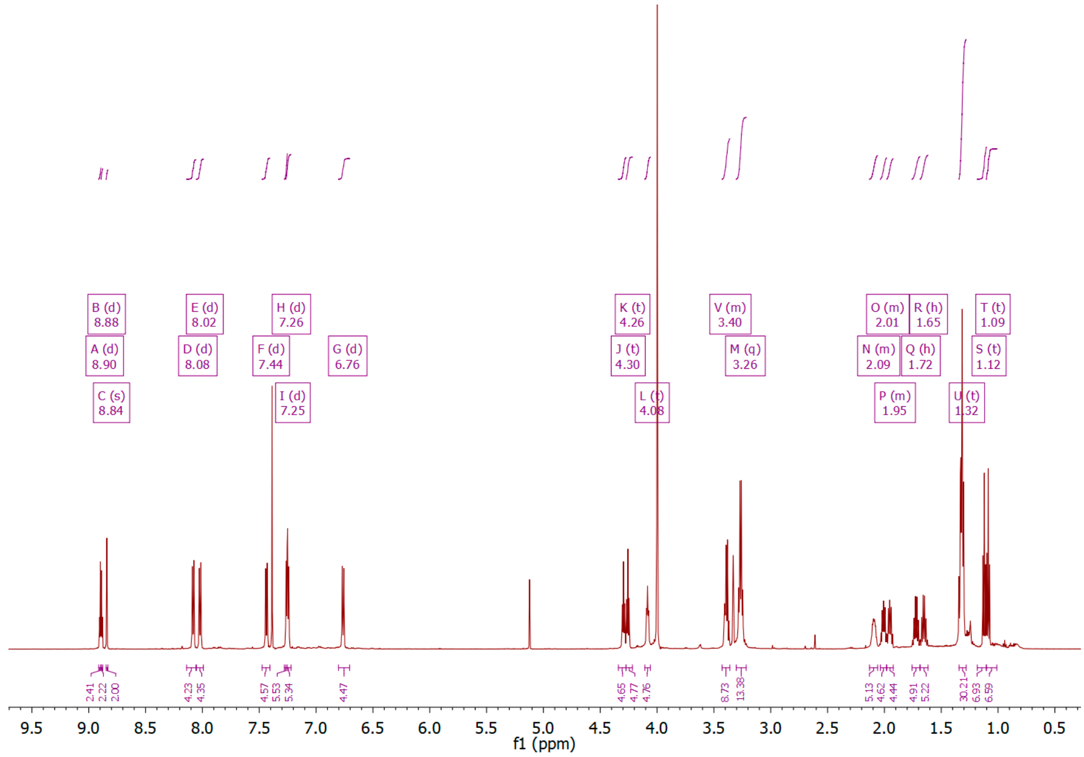

Figure S5. ^{1}H NMR spectrum of **Zn-1** (20% MeOD in $CDCl_3$).

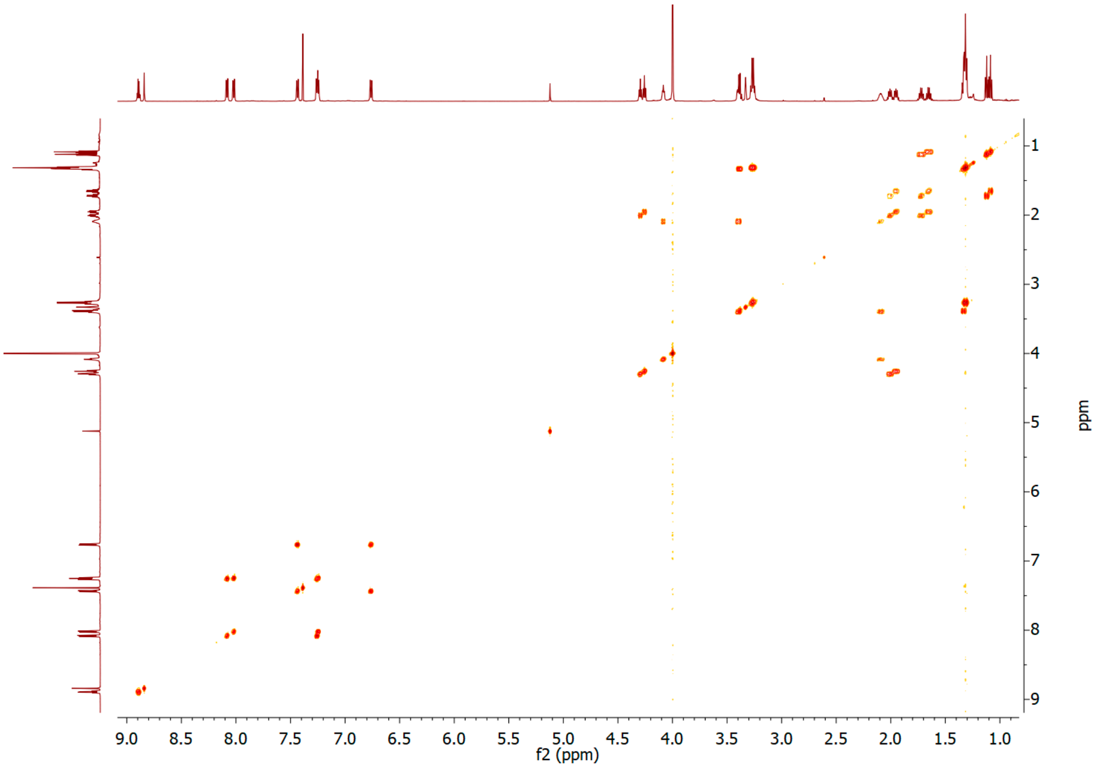

Figure S6. ^{1}H-^{1}H COSY spectrum of **Zn-1** (20% MeOD in $CDCl_3$).

UV-vis spectra of the dicationic compounds

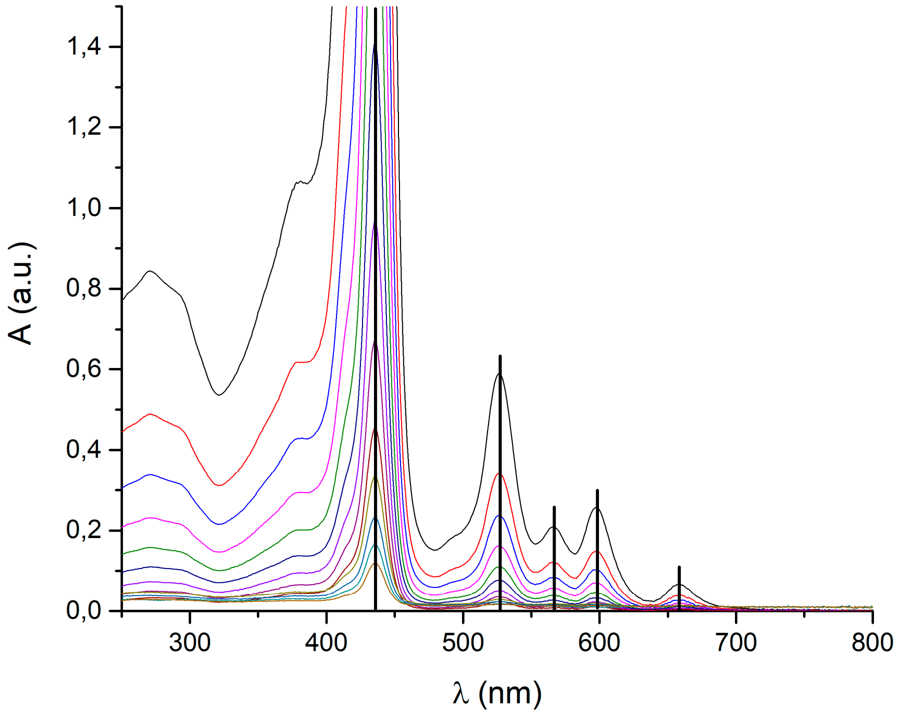

Figure S7. A series of UV–Vis spectra of **2H-1** in $CHCl_3$ ($C = 2.6\times10^{-5}$–2.4×10^{-7} M).

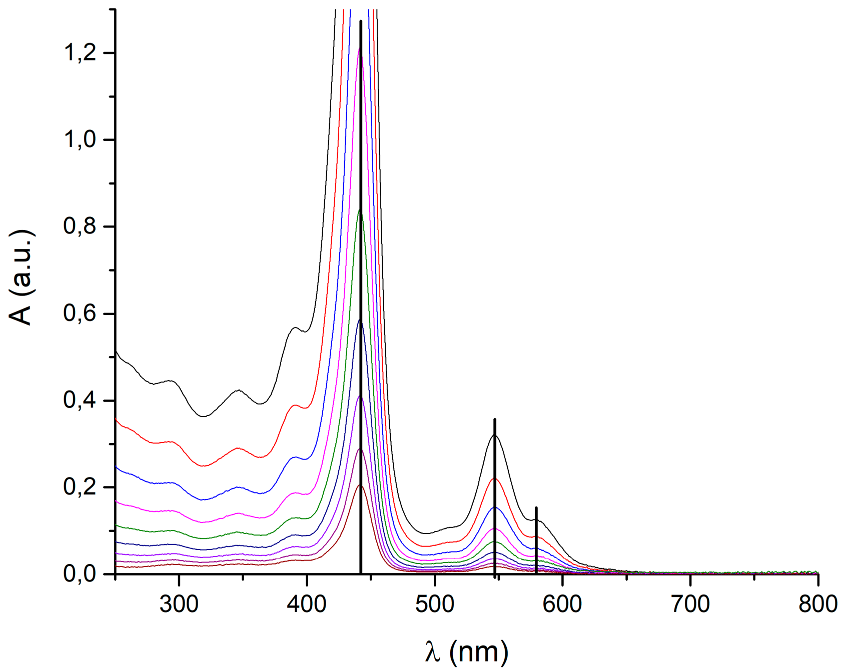

Figure S8. A series of UV–Vis spectra of Ni-1 in CHCl3 (C = 2.1×10–5–1.4×10–6 M).

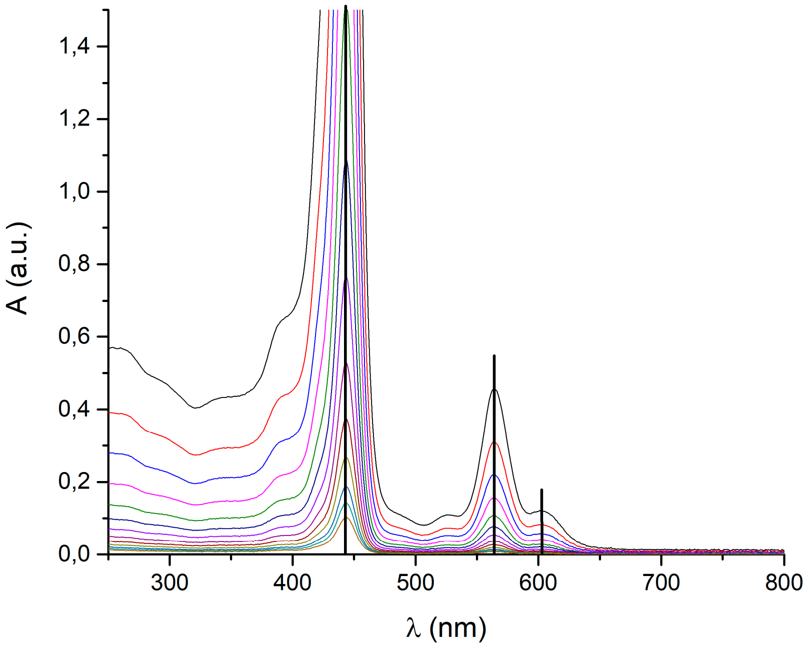

Figure S9. A series of UV–Vis spectra of Zn-1 in CHCl3 (C = 1.9×10−5–1.8×10−7 M).
